# Prevalence of and factors influencing postnatal depression in a rural community in South Africa

**DOI:** 10.4102/phcfm.v7i1.874

**Published:** 2015-11-24

**Authors:** Ethelwynn L. Stellenberg, Johanna M. Abrahams

**Affiliations:** 1 Division of Nursing, Stellenbosch University, South Africa

## Abstract

**Background:**

Knowledge about postnatal depression (PND) and associated risk factors which influence the development of PND is vital for early detection, intervention and prevention.

**Setting:**

The study was conducted in primary health care clinics (PHC) in the Witzenberg subdistrict, a rural community in South Africa.

**Objectives:**

Objectives of this study were to determine the prevalence of PND and to identify the contributing risk factors associated with PND.

**Methods:**

A descriptive cross sectional research design with a quantitative approach was applied. The target population was mothers, 18 years and older. A convenience sampling method was used to select a sample of 159 (10%) from a population of 1605 live births. The Edinburgh Postnatal Depression Scale (EPDS) and Beck Depression Inventory (BDI), two validated self-rating questionnaires, including a questionnaire based on demographical, psychosocial and obstetrical data, were applied. The data was analysed using various statistical tests to determine statistical associations between variables using a 95% confidence interval.

**Results:**

PND was a serious health problem with 50.3% of the mothers who suffered from PND. A BDI analysis showed that of the participants who had PND, 28.8% was severe, 48.8% moderate and 22.5% mild. Factors influencing the development of PND included most participants (63.5%) were unmarried, 61.3% were unemployed and the majority (53.8%) had a history of a psychiatric illness. Significant associations between PND and unplanned and unwelcome babies (*p* < 0.01); partner relationship (*p* < 0.01); were identified.

**Conclusion:**

Prevention, early detection, appropriate referral and treatment of PND are critical in managing maternal, child and family well-being.

## Abstrak

Prévalence de, et facteurs qui influencent la dépression postnatale dans une communauté rurale d’Afrique du Sud.

**Contexte:**

Une connaissance de la dépression postnatale (DPN) et des facteurs de risque connexes qui influencent le développement de la DPN est essentielle pour la détection précoce, l’intervention et la prévention.

**Lieu:**

L’étude a été menée dans des cliniques de santé primaire (CSP) du sous-district de Witzenberg, une communauté rurale d’Afrique du Sud.

**Objectifs:**

Cette étude avait pour objet de déterminer la prévalence de la DPN et d’identifier les facteurs de risques liés à la DPN.

**Méthodes:**

On a utilisé un plan de recherche transversal descriptif avec une approche quantitative. La population visée était des mères de 18 ans et plus. On a fait appel à une méthode d’échantillonnage représentative pour sélectionner un échantillon de 159 (10%) personnes sur une population de 1605 naissances vivantes. On a appliqué l’Echelle de Dépression Postnatale d’Edinburgh (EDPE) et l’Inventaire de Dépression de Beck (IDB), deux questionnaires autoévalués et validés, comprenant un questionnaire basé sur des données démographiques, psychosociales et obstétriques. On a analysé les données au moyen de différents tests statistiques pour déterminer les associations statistiques entre les variables avec un intervalle de confiance de 95%.

**Résultats:**

La DPN est un grave problème de santé avec 50.3% des mères qui en souffrent. Une analyse IDB a montré que parmi les participantes souffrant de DPN, 28.8% étaient sévèrement affectées, 48.8% modérément et 22.5% légèrement. Les facteurs influençant le développement de la DPN étaient que la plupart des participantes (63.5%) n’étaient pas mariées, 61.3% étaient au chômage et la majorité (53.8%) avait des antécédents de maladie psychiatrique. On a aussi remarqué des liens entre la DPN et les bébés non planifiés et non désirés (*p*< 0.01); et la relation des partenaires (*p* < 0.01).

**Conclusion:**

La prévention, la détection précoce, l’orientation vers le service et le traitement appropriés de la DPN sont critiques pour la gestion du bien-être de la mère, de l’enfant et de la famille.

## Introduction

### Social value

Depression is associated with substantially reduced quality of life and functional capacity for the affected woman with PND.^1^ Poor maternal mental health is especially problematic for the infants of these mothers. The investigation of the prevalence and the factors contributing to PND was fundamental in contributing to the achievement of the Millennium Development Goals, specifically in improving maternal health and reducing child mortality.^2^ The lack of scientific evidence about the prevalence and factors contributing to PND in the Witzenberg subdistrict, Western Cape, South Africa made it essential that this problem was investigated scientifically. Ultimately, this study may contribute to early detection and management of PND and the improvement of the mental health services in the rural community.

Depression is the leading cause of disease burden for women in both high-income and low- and middle income countries.^3^ Despite being a leading cause of disease burden for women, mental health is still the stepchild of health services and even more so in the rural areas.^4^

Postnatal depression (PND) is a severe condition, which can be described as ‘a thief who steals maternity’.^5^ The prevalence of PND is currently considered to be 10% – 15%, but is still increasing and becoming a serious public health problem.^6,7^ In a study conducted in a South African peri-urban settlement, Khayelitsha, a prevalence rate of 34.7% of mothers at two months’ postnatal suffer from PND.^8^ PND affects approximately 500 000 mothers in the United States each year, and about one in five mothers may be affected by a major mental disorder in the first 12 months following childbirth.^9^ The seriousness of this condition is further shown in an incident as reported in the IOL News, London, that a 35-year-old woman with PND killed her two babies, a 10-week-old son and 14-month-old daughter.^10^

### Scientific value

PND is a universal and serious mood disorder that occurs within a few weeks after birth and causes considerable risks to the mother, the developing child and the entire family.^9^ The derivations of PND can sometimes be visible during the postnatal period, sometimes referred to as puerperium, but are frequently only fully recognisable months later in the first year after birth. This depression may last for weeks, months or even years after origination.^11^ Therefore, if PND is left untreated, it may lead to affective, cognitive, behavioural and physical problems. This may not only have an extremely negative effect on the mothering role and mother-child relationship, but on the total existence of the affected mother's family.^12^

Almost 10% of all postnatal mothers will acquire a depressive disorder.^13^ It was further shown that it is most likely to occur in 16% of women during the postpartum period.^14^ Mental illnesses, especially major depression, add extensively to the non-fatal burden of disease and is the second leading cause of mental disabilities. However, in South Africa the ratio between psychiatrist and patient and/or client is 1:357 142 and psychologist and patient and/or client is 1:312 500,^15^ making mental illness one of the most neglected illnesses in South Africa. The Annual Health Status Report identified that in the Witzenberg subdistrict there was a marked increase in mental health cases from 347 in 2001 to 491 in 2006.^16^

Depression, anxiety, stressful life events and low levels of social support were identified as key maternal risk factors in studies conducted in Khayelitsha.^14,17^ Nearly one third of employed participants (32.7%) in a study stated substantial depressive symptoms at four months postnatal.^18^ Thus, it is evident that PND will influence the mother's capability to care for her baby.^19^

Maternal age is related to PND symptoms but lower socio-economic and educational levels are added risk factors.^20^ Symptoms of depression are substantially higher amongst mothers with lower educational levels who are unmarried and poor.^18^ PND has been found to be higher in socio-economically disadvantaged areas in SA, as shown in a study in Khayelitsha, in which the PND rate is as high as 34.7%.^8^

The levels, however, in teenage mothers have been reported to be as high as 26%.^6^ The incorporation of many factors such as integration of biochemistry, hormonal functioning, genetic history and psychosocial factors such as stressful life events concede the potential for the occurrence of PND.^6^

### Conceptual theoretical framework

The conceptual theoretical framework, which guided this study, is based on the clinical or medical model, bio-psychosocial model, holistic approach of nursing, and the following psychosocial theories of depression: psychoanalytic theory, object loss theory, learned helplessness theory and the cognitive theory. These models and theories were significant for this study because it is imperative for health care workers to understand depression, the origin thereof, and what the possible causes and consequences are in order to understand and manage clients most effectively.

### Aim and objectives

The objectives of this study were to determine the prevalence of PND and to identify the contributing risk factors associated with PND in the Witzenberg subdistrict of the Western Cape, South Africa.

## Research methods and design

### Study design

A cross sectional descriptive research design with a quantitative approach was applied in this study.

### Setting

The study was conducted in four of the eight clinics in the Witzenberg subdistrict, with a population of approximately 115 946 in 2011.^21^ The subdistrict under study included the five towns of Ceres, Prince Alfred Hamlet, Op-Die-Berg, Wolseley and Tulbagh, with a rural predominance and a high degree of poverty.^22^

### Study population sampling strategy

The target population included mothers (*N* = 1605) who gave birth during one calendar year and were resident in the Witzenberg subdistrict of the Western Cape. Simple random sampling was used to select four clinics (50%), two clinics in the lower socio-economic areas and two clinics in the middle socio-economic areas. Mothers (≥ 18 years old) were selected when they brought their babies to the clinics for immunisations at 6-, 10- or 14 weeks. The researcher addressed all the mothers in a group and explained the purpose of the study and requested if there were any mothers who would willingly participate. Thus, 159 mothers, approximately 10% of the target population, were selected.

### Data collection

Data were collected over a period of four months, once a week at the individual postnatal clinics identified for the purpose of the study. Data collection was done by the researcher with the help of two trained field workers with a nursing background and who were fluent in Afrikaans, English and Xhosa. The field workers were trained beforehand in the interpretation of the different questionnaires and the process of data collection. Their role was to assist with interpretation. The questionnaire was self-administered and where participants had a problem understanding any question, it was explained by the researcher or the trained field worker.

The questionnaire, based on the literature and clinical experience of the PI, was designed and administered to determine the demographical data and psychosocial risk factors of the participants. The questions specifically included aspects about: maternal age, marital status, level of education, pregnancies and children, mode of delivery, psychosocial factors, psychiatric history and social support.

The Edinburgh Postnatal Depression Scale (EPDS) was used to identify postnatal depression. The questionnaire is easy to administer and takes approximately five minutes to complete and is generally accepted by mothers after birth. The questionnaire consists of ten questions specifically about the participant's feelings over the previous seven days, including the day she was interviewed.^23^

The Beck Depression Inventory (BDI) was also used in this study to determine the severity of PND. The BDI (BDI-II),^24^ created by Dr Aaron T. Beck, is a 21-question multiple-choice self-report inventory and is one of the most widely used instruments for measuring the severity of depression. Each question consists of a set of at least four possible choices, with values ranging in intensity from 0–3.^25^ No values were visible on the participant's copy to minimize the risk of the participant manipulating the outcome, which may lead to answers being chosen because of its specific value. The classification to determine the severity of PND was as follows:

- Score < 15: Mild Depression
- Score 15–30: Moderate Depression
- Score > 30: Severe Depression

## Reliability and validity

The EPDS and BDI are two validated, self-rating questionnaires. The authors^23^ of the EPDS have shown that the 10-item Postnatal Depression Scale had satisfactory validity, split-half reliability and that it is also sensitive to changes in the severity of depression over time. The BDI is a well-known screening instrument in the mental health field. It is recommended for research, as well as in the clinical setting for its reliability and validity. The reliability and validity was further ensured through the use of experts in the fields of statistics, nursing and research methodology. The pilot study also added to the validity and reliability because all three instruments were pre-tested and rectified, where required, after the pilot study.

### Data analyses

All data were analysed and interpreted with the support of a qualified statistician. A computer software program, STATISTICA,^26^ was used to analyse the data. Data were expressed in frequencies, tables and histograms. The relationship between continuous response variables and nominal input variables was analysed using analysis of variance (ANOVA). Statistical associations and significance between variables were determined by using a 95% confidence interval, with a significance level of (*p* ≤ 0.05).

## Ethical considerations

The researchers obtained written permission from the Committee for Human Science Research at the Faculty of Medicine and Health Sciences at Stellenbosch University (ref. N09/11/336). The researchers also received written consent from the Research Coordinating Committee of the Department of Health (RP16/2010), Provincial Government of Western Cape.

### Potential benefits and hazards

The researcher explained the study to all the participants individually and emphasised that their participation was voluntary, and informed written consent was obtained from all participants. The risk foreseen was the sudden breakdown of participants during the interview that may then have required counselling.

### Recruitment procedures

Participants were informed that participation was voluntary and that they had a choice to withdraw from the study at any stage and that their decision would not influence any further service rendered to them.

### Informed consent

Voluntary written informed consent was obtained from individual participants after providing information about the research. The ethical implications, as well as the aim and objectives for the study, were explained to each participant personally.

### Data protection

Data obtained from the study was coded to protect the identities of the participants and to ensure confidentiality, anonymity and privacy concerning all information. The data is stored in a secure locked cabinet providing access to the researchers only.

## Results

A total of 159 participants participated in the study of which 80 (50.3%) had PND as analysed using the EPDS test (Tables 1 and 2). The BDI test showed that of the participants (80) who had PND, 23 (28.8%) had severe depression, 39 (48.8%) moderate and 18 (22.5%) mild depression (Table 3).

**TABLE 1:** Summary of demographical and obstetrical factors as classified according to the Edinburgh Postnatal Depression Scale test.

Demographical and obstetrical factors	All *n* = 159 (%)	Not depressed *n* = 79 (%)	Depressed *n* = 80 (%)	*p*-value
**Marital status**				
Married	29 (18.2)	11 (13.9)	18 (22.5)	*p* = 0.17
Unmarried	102 (64.2)	57 (72.2)	45 (56.3)	
Living together	26 (16.4)	10 (12.7)	16 (20.0)	
**Teenagers**				
Mode of delivery	38 (23.9)	19 (24.1)	19 (23.8)	*p* = 0.20 *p* = 0.28
Normal births	133 (83.7)	62 (78.5%)	71 (88.7)	
Caesarean	25 (16.3)	16 (20.5%)	9 (11.3)	
**Postnatal period**				
Postnatal period 4	53 (33.3)	32 (40.5)	21 (39.6)	*p* = 0.05
Postnatal period: 10 weeks	52 (32.7)	22 (27.8)	30 (57.7)	
Postnatal period: 14 weeks	54 (34.0)	25 (31.6)	29 (53.7)	
**Employment**				
Employed	64 (40.3)	33 (41.8)	31 (38.8)	*p* = 0.04
Unemployed	95 (59.7)	46 (58.2)	49 (61.3)	
**Income**				
No income	40 (25.1)	21 (26.6)	19 (23.8)	*p* = 0.57
Single income	65 (40.9)	37 (46.8)	28 (35.0)	
Family income	54 (34.0)	21 (26.6)	33 (41.3)	

**TABLE 2:** Factors influencing the development of postnatal depression as classified according to the Edinburgh Postnatal Depression Scale test.

Factors influencing development of PND	All *n* = 159 (%)	Not depressed *n* = 79 (%)	Depressed *n* = 80 (%)	*p*-value
**Psychiatric history**				
Psychiatric self- history	65 (40.9)	22 (27.8)	43 (53.8)	*p* < 0.01 *p* = 0.24
Psychiatric family history	138 (86.8)	71 (89.9)	67 (83.9)	
**Life events**				
Major life events	67 (42.1)	28 (35.4)	39 (48.8)	*p* = 0.10
Minor life events	39 (24.5)	23 (29.1)	16 (20)	
No life events	47 (29.6)	26 (32.9)	21 (26.3)	
Major and minor	5 (3.1)	2 (2.5)	3 (3.8)	
Minor and minor	1 (0.6)	0 (0.0)	1 (1.3)	
**Partnership**				
Unsatisfactory partnership	65 (40.9)	21 (26.6)	44 (55.0)	*p* < 0.01
Satisfactory partnership	94 (59.1)	58 (73.4)	36 (45.0)	
**Family and social support**				
Have family and social support	123 (77.4)	71 (89.9)	52 (65.0)	*p* < 0.01
Have no family and social support	36 (22.6)	8 (10.1)	28 (35.0)	
**Baby factors**				
Unplanned babies	96 (60.4)	54 (68.4)	42 (52.5)	*p* = 0.03
Planned babies	46 (28.9)	21 (26.6)	25 (31.3)	
Unwelcome babies	13 (8.1)	3 (3.8)	10 (12.5)	
Unwelcome sex of baby	4 (2.5)	1 (1.3)	3 (3.8)	

PND, postnatal depression.

**TABLE 3:** Factors influencing the development of postnatal depression showing the severity of depression levels as classified according to the Beck Depression Inventory test.

Factors influencing development of PND	Depression *n* = 80 (50.3%)	Mild *n* = 18 (22.5%)	Moderate *n* = 39 (48.8%)	Severe *n* = 23 (28.8%)	*p*-value
**Teenager**					
Yes	19 (23.8)	6 (33.3)	8 (20.5)	5 (21.7)	*p* = 0.56
No	61 (76.2)	12 (66.7)	31 (79.5)	18 (78.3)	
**Marital status**					
Unmarried	45 (56.3)	13 (72.2)	19 (48.7)	13 (56.5)	*p* = 0.03
Married	18 (22.5)	1 (5.6)	15 (38.5)	2 (8.7)	
Living together	16 (20)	4 (22.2)	5 (12.8)	7 (30.4)	
Widow	1 (1.3)	0 (0)	0 (0)	1 (4.4)	
**Mode**					
Normal	71 (88.7)	17 (94.4)	33 (84.6)	21 (91.3)	*p* = 0.47
Caesarean	9 (11.3)	1 (5.6)	6 (15.4)	2 (8.7)	
**Employment**					
Employed	31 (38.8)	9 (50)	15 (38.5)	7 (30.4)	*p* = 0.44
Unemployed	49 (61.2)	9 (50)	24 (61.5)	16 (69.6)	
**Income**					
None	19 (23.8)	5 (27.8)	8 (20.5)	6 (26.1)	*p* = 0.98
Single	28 (35)	7 (38.9)	13 (33.3)	8 (34.8)	
Family	33 (41.3)	6 (33.3)	18 (46.2)	9 (39.1)	
**Psychiatric history: Family**					
Family: Yes	13 (16.3)	3 (16.7)	5 (12.8)	5 (21.7)	*p* = 0.06
Family: No	67 (83.7)	15 (83.3)	34 (87.2)	18 (78.3)	
**Psychiatric history: Self**					
Self: Yes	43 (53.8)	7 (38.9)	19 (48.7)	17 (73.9)	*p* ≤ 0.01
Self: No	37 (46.2)	11 (61.1)	20 (51.3)	6 (26.1)	
**Life events**					
Major	39 (48.7)	4 (22.2)	23 (59)	12 (52.2)	*p* = 0.01
Minor	16 (20)	5 (27.8)	4 (10.3)	7 (30.4)	
MN and MJ	3 (3.8)	1 (5.6)	0 (0)	2 (8.7)	
MN and MN	1 (1.3)	1 (5.6)	0 (0.0)	0 (0.0)	
No life event	21 (26.2)	7 (38.9)	12 (30.8)	2 (8.7)	
**Partner relationships**					
Unsatisfactory	44 (55)	3 (16.7)	20 (51.3)	21 (91.3)	*p* < 0.01
Satisfactory	36 (45)	15 (83.3)	19 (48.7)	2 (8.7)	
**Baby factors**					
Unplanned	42 (52.3)	11 (61.1)	20 (51.3)	11 (47.8)	*p* < 0.01
Planned	25 (31.3)	5 (27.8)	14 (35.9)	6 (26.1)	
Unwelcome	10 (12.5)	1 (5.6)	4 (10.3)	5 (21.7)	
Sex of baby unwelcome	3 (3.8)	1 (5.6)	1 (2.6)	1 (4.4)	
**Family and/or social support**					
Yes	52 (65.0)	15 (83.3)	28 (71.8)	9 (39.1)	*p* < 0.01
No	28 (35.0)	3 (16.7)	11 (28.2)	14 (60.9)	

## Demographical and obstetrical factors

### Marital status

Aspects about the marital status and depression are shown in Table 1. The majority of the participants 102 (64.2%) were unmarried and 26 (16.4%) were living with a partner (Table 1). The maternal age of participants indicated that 38 (23.9%) were teenagers, age 18–19 years, of which 19 (50%) had PND. The BDI test revealed that amongst the teenagers, 21.7% had severe depression, 20.5% had moderate and 33.3% had mild depression (Table 3).

A bivariate analysis showed that 45 (56.3%) of the participants who were unmarried, 18 (22.5%) who were married and 16 (20%) who were living with a partner suffered from depression (Table 1).

BDI inventory analysis results showed that of the unmarried participants 56.5% had severe depression (Table 3).

### Pregnancies

Most of the participants 71 (44.7%) had only had one (1) pregnancy followed by 39 (24.7%) who had had two (2) pregnancies. No significant results were obtained between depression and pregnancies.

### Mode of delivery

The results showed that of the participants (*n* = 80) with PND 88.7% had normal births (Table 1). According to the BDI test 91.3% had severe depression and 84.6% moderate depression (Table 3).

### Postnatal period 6–10–14 weeks

Table 1 shows the number of participants at the various time intervals of 6–10–14 weeks. The analysis further showed that 39.6%, 57.7% and 53.7% of the participants had PND at the particular week intervals 6–10–14 weeks respectively. A significant statistical association (*p* = 0.02) was obtained between the 6-week and 10th week periods with the Bonferroni post-hoc test. A further significant association was identified between the EPDS test and postnatal period (*p* = 0.05) (Table 1).

### Employment

Results showed that 95 (59.7%) of the participants were unemployed of which 49 (61.3%) of the participants with PND were unemployed. A statistical association was identified between employment and the EPDS test (*p* = 0.04) applying the Mann-Whitney *U*-test (Table 1). According to the BDI test 30.4% had severe and 38.5% had moderate depression (Table 3).

### Income

The results showed that 119 (74.9%) of the participants have an income whilst 40 (25.1%) have no income. A further analysis showed that of the number of participants who suffered from PND 23.8% of these participants received no income (Table 1). The BDI test revealed that the participants with only a family income 46.2% had moderate depression whilst 39.1% had severe depression. No statistical significant difference was shown between the depression and income (*p* = 0.98) (Table 3).

## Factors influencing the development of postnatal depression

### Psychiatric history

Table 2 shows that 65 (40. 9%) had a history of a psychiatric illness, of which 53. 8% of the participants who had PND (*n* = 80) had a history of a psychiatric illness. Using the Mann-Whitney *U*-test a statistical significant association was identified between psychiatric history of the self and depression (*p* < 0.01).

The results further showed that 83.7% of the participants with PND (*n* = 80) had no family history of psychiatric illnesses. The BDI test showed that participants with no family history 78.3% had severe depression, 87.2% moderate and 83.3% mild depression (Table 3).

### Life events

The results show that 67 (42.1%) of the participants had experienced a major life event which excluded the delivery of a baby, which is a natural event. In addition, results illustrated that of the 80 participants who suffered from PND, 48.7% experienced major life events, 20% a minor event and 21 (26.3%) had no life event experiences (Table 2). According to the BDI test, participants who had a major life event 59% had moderate depression and 52.2% severe depression (Table 3).

Furthermore, the study also shows a statistical significant difference between depression and life events using the Chi square test (df = 8) = 19.95, (*p* = 0.01) (Table 3).

### Partner relationship

Table 2 shows that 65 (40.9%) of participants were in an unsatisfactory partner relationship, of which 55% of the 80 participants who suffered from PND, were in unsatisfactory relationships with their partners. The BDI test shows that 91.3% of the participants had severe depression and 51.3% moderate depression. A statistical significant difference between partner relationship and the EPDS test applying the Mann-Whitney *U*-test was identified (*p* < 0.01) (Table 2) and between partner relationship and depression applying the Chi square test (df = 2) = 26.25 (*p* < 0.01) (Table 3).

### Family and social support

The majority of the participants 123 (77.4%) received family and social support (Table 2). However, of the participants who suffered from PND 35% received no family or social support.

The Levene's test for homogeneity of variances effect also showed a significant difference between family or social support and the EPDS test (*p* < 0.01) (Table 2).

A statistical significant difference was shown between family and social support and depression using the Mann-Whitney *U*-test (*p* < 0.01). According to the BDI test, participants who received no family or social support (60.9%) had severe depression and 11 (28.2%) had moderate depression (Table 3).

### Baby factors

The baby factors are defined as those factors associated with the baby which may have contributed to PND, namely unplanned, planned, unwelcome baby or an unwelcome sex of the baby. The majority of participants 96 (60.4%), as shown in Table 2, had unplanned babies. The results further showed that of the 80 participants who suffered from PND, 52.5% were unplanned pregnancies and 31.3% were planned babies, 12.5% were unwelcome and 3.8% were unwelcome sex of the babies. The Kruskal Wallis test further showed a significant difference between baby factors and EPDS test (*p* = 0.03) (Table 2) and between baby factors and depression (*p* < 0.01) (Table 3). However to guard against Type 1 error, a post-hoc analysis was done by applying the Bonferoni test using the BDI variable specifically to determine specific statistical differences between the baby factors. The Bonferoni test revealed a statistical significant difference between unplanned and unwelcome babies (*p* < 0.01) and between planned and unwelcome babies (*p* < 0.01). The Bonferoni test was also applied using the EPDS variable to determine any statistical differences between the baby factors but no significant associations were shown.

## Discussion

### Key findings

The results of this study revealed that 50.3% of the participants had PND with 48.8% of the participants with moderate PND in the Witzenberg subdistrict, a rural area of South Africa. Most participants who developed PND were affected by more than one preventable risk factor. The dominant factors contributing to PND in this rural environment were that most participants 64.2% were unmarried, of which 56.3% had PND, 55% of participants with PND were in unsatisfactory relationships, and the majority 60.4% had unplanned babies, of which 52.5% of all participants with PND were unplanned. Results have shown that teenage pregnancies in this rural environment could be problematic as 23.8% of the participants with PND were teenagers. Compounded further is that more than half of the participants 53.8% who had PND had a history of a psychiatric illness and had experienced a major life event 48.7%. This rural study also showed that 59.7% of the participants were unemployed with the majority 61.3% of the participants with PND being unemployed.

### Discussion of key findings

The results of this study do not correspond to the worldwide prevalence rate of 12% – 15%, as found in many of the community surveys of postnatal depression, but is similar to a study which states that some studies have identified PND up to 60%.^27^ The study revealed that 55% of the participants with PND were in an unsatisfactory relationship with their partners with a statistical significant difference between partner relationship and depression (*p* < 0.01). These results between partner relationship and PND are aligned with results of a study which found that poor quality of relationships may increase PND.^9^ The lack of partner support, or closeness, or partner abuse is found in nearly one in three of the women with PND.^28^ Therefore, it remains important to have a spouse or partner for protection against developing PND.^29^Teenage pregnancy contributes to the development of PND.^30^ This is substantiated further in a study which confirms that pregnancy at a very young age may increase the prevalence of PND.^9^ It is thus a concern that in this study 38 (23.9%) of participants were teenagers, 18–19 years of age of which 19 (50%) had PND. In this particular region under study, 142 babies were born to teenagers below the age of 18 years over a period of one year, April 2014 − March 2015,^31^ which increases the risk for the development of PND amongst teenagers in this region.

Most of the participants (52.5%) with PND had unplanned babies. These results are aligned with a study^8^ which identified that unplanned pregnancies contribute to maternal depression.

Poor maternal mental health is especially problematic for the infants of these mothers as it reduces the caregiver's sensitivity and responsiveness at a time when a child is entirely dependent on his or her mother's well-being.^32^

### Strengths and limitations

The research was conducted in a rural subdistrict of the Western Cape, South Africa, which included five towns. A quantitative study was conducted using international validated instruments, however a phenomenological qualitative research may provide in-depth experiences about specific factors that influence the development of PND. A limitation of the study was that a convenient sample was drawn of participants who were available, met the criteria and gave voluntary consent to participate in the study. The research was conducted only in the public health services and participants were predominantly of a low socio-economic level.

### Implications or recommendations

The results of this study revealed that the risk factors as discussed and identified in the Witzenberg subdistrict contributed to the development of PND. In most cases, PND can be prevented and, if diagnosed early, the level of severity and consequences of the problem may be curtailed. The recommendations are thus based on the scientific evidence obtained in this study with specific reference to preventative and promotive measures.

### Early assessment

Early and holistic assessment of the pregnant women during the antenatal period should be done to identify any risk factors and clinical symptoms of depression which may necessitate early referral and/or treatment.

### In-service and continuous professional development

Continuous professional development and in-service training of midwives and primary health care practitioners are recommended. Nurses should be empowered to take the appropriate measures when factors or any symptoms are identified, as supported by Tammentie et al.^33^

### Nutritional supplements

The nutritional status of the patient is specifically important during the antenatal visits as the midwife needs to ensure that the mother understands the importance of a balanced diet and taking her supplements, namely ferrous sulphate and folic acid tablets. There is a strong relationship between iron status and PND.^34^

### Prevention of teenage pregnancies

All stakeholders of the communities, including churches, non-governmental organisations, schools, the South African Police Services and the Departments of Education and Social Development and Health should apply a unified approach in combating social pathologies such as sexual abuse and prostitution, which contribute to the high incidence of pregnancy.

### Role of the department of health

Woman's health is a priority and should be addressed as a priority to ensure healthy women and babies. Standard practices should include prenatal and postnatal classes for mothers and fathers. User-friendly clinics especially designed for teenagers have become essential.

Comprehensive school health programmes must be fully implemented at all schools, such as the ‘Health Promoting Schools Initiative’, and should include a healthy sexuality programme.

### Further research

Qualitative research is recommended in the low-income rural areas in South Africa to obtain a better understanding of the communities’ understanding of mental distress and their mental health needs. In addition, as described, a phenomenological qualitative research may provide in-depth experiences about specific factors that influence the development of PND.

## Conclusion

The study has shown an extremely high prevalence of risk factors for the development of PND. Prevention, early detection, appropriate referral and treatment of PND are thus critical in managing maternal, child and family well-being.
